# Demographic and ecological niche dynamics of the Vietnam warty newt, *Paramesotriton deloustali*: Historical climate influences

**DOI:** 10.1371/journal.pone.0290044

**Published:** 2023-08-18

**Authors:** Dung Van Tran, Thinh Tien Vu, Kazumi Fukutani, Kanto Nishikawa

**Affiliations:** 1 Graduate School of Human and Environmental Studies, Kyoto University, Kyoto, Japan; 2 Wildlife Department, Vietnam National University of Forestry, Ha Noi, Vietnam; 3 Graduate School of Global Environmental Studies, Kyoto University, Kyoto, Japan; Guangxi University, CHINA

## Abstract

Quaternary climatic cycles strongly affected the genetic diversification and ranges of organisms, shaping current genetic structures and distribution patterns. Urodeles provide ideal examples for exploring these dynamics over time and across space. In this study, we integrated a phylogeographic approach and ensemble species distribution modeling (eSDM) to infer the historical demography and distribution patterns of the Vietnam warty newt, *Paramesotriton deloustali*. Mitochondrial data revealed two groups, West and East, which diverged approximately 1.92 million years ago (Mya). Diversification was likely driven by change in the climate during early stages of the Pleistocene, with increasing monsoon and drought intensities. Biogeographic analysis indicated that the newt’s current distribution formed as a result of vicariance events. In addition, the two groups occupy distinct ecological niches. Demographic reconstruction showed signs of expansion in the effective population sizes of the two major groups beginning around 0.11 and 0.15 Mya, respectively. However, eSDM showed fluctuating predicted distributions during the last interglacial, last glacial maximum, mid-Holocene, and present. Mountain systems in northern Vietnam are likely to have served as climatic refuges and to have played a crucial role in safeguarding species from the effects of climate change.

## Introduction

The factors underlying the distribution patterns and divergence of organisms are a central topic in biogeography [1], and understanding them is crucial to developing sustainable management and conservation strategies [2, 3]. The geographic distribution of species is controlled by a combination of abiotic and biotic factors over species’ evolutionary histories [4]. As the distribution of a species is affected by numerous processes with complex patterns across time and space, understanding its driving factors remains a major challenge [5]. The recent integration of phylogeography with species distribution modeling (SDM) provides a robust approach to exploring population dynamics and historical distribution processes, revealing the drivers underlying the current ranges of species [6–10]. Phylogeography can be used to illustrate the spatial genetic diversity and structure of species as well as to estimate demographic dynamics [6, 8, 11], while SDM provides insights into the relationships of species with environmental space, ecological interactions among species, and the process of speciation [9, 12, 13].

Northern Vietnam is located in Southeast Asia, a global biodiversity hotspot [14, 15]. The geology and environment of northern Vietnam are highly complex due to its location at the convergence of the tropical and subtropical zones and biotic interactions among three biogeographic units: Indochina, south China, and coastal Indochina [16]. This area is dominated by the Hoang Lien Son Mountain Range (the southeasternmost extension of the Himalaya Range), karst formations, and the Red River [16]. The climate is highly seasonal, with considerable variation in temperature and rainfall. In addition, the area is affected by cold and arid air delivered by the northeast monsoons from the Tibetan Plateau [16]. During the Pleistocene, cooler and drier climatic events greatly impacted northern Vietnam, despite the absence of glaciers [17, 18]. The majority of lowland areas were likely covered by wetlands and grasslands rather than tropical rainforests during the last glacial maximum (LGM) [17, 19]. Multiple studies have demonstrated that the genetic divergence, population demographics, and current ranges of a variety of organisms have been significantly influenced by climatic oscillations during the Quaternary period [20–26]. However, species may exhibit diverse responses to ensure their survival through significant climate changes [23].

Urodeles are excellent examples for exploring the demographic dynamics and historical ranges of organisms over time and across space due to their long evolutionary histories, low dispersal abilities, and strong environmental constraints [27]. Historical climate significantly shapes the geographic distributions and genetic diversification of salamanders and newts [10, 13, 28–31]. The Vietnam warty newt (*Paramesotriton deloustali*, Caudata: Salamandridae) provides an ideal model for assessing the effect of Quaternary climate on the demographic and range dynamics of organisms. This species occurs widely in northern Vietnam [32, 33] and southern China [34], with a wide elevational range of 200–1900 m asl [32, 35]. Currently, *P*. *deloustali* is classified as “Least Concern” on the International Union for Conservation of Nature (IUCN) Red List [32]. Similar to other warty newts, *P*. *deloustali* prefers cool montane streams in closed-canopy evergreen forest habitats and possesses limited physiological tolerance to extremely hot and dry environments [35]. However, the relationships between genetic structure patterns and dynamic environmental characteristics of this species have not yet been studied.

The dynamics of various species during the glacial period in Indochina have been elucidated in previous studies, including the Ornate chorus frog, *Microhyla fissipes* [36]; Giant spiny frog, *Quasipaa spinosa* [37]; forest-dwelling murine rodents of the genus *Leopoldamys* [38]; and the Fishing cat, *Prionailurus viverrinus* [39]. Despite interest in clarifying the responses of tropical warty newts to past climate variation, a shortage of research in this area persists. Here, we integrated mitochondrial DNA (mtDNA) sequence data with environmental data in SDMs to reveal the historical demographic and distribution patterns of *P*. *deloustali*, the southernmost species of genus *Paramesotriton*, at both the species and population levels. We highlighted the role of the Quaternary climate in driving the genetic diversification, demographic structure, and paleodistribution of the species. Specifically, we assessed the genetic structure of *P*. *deloustali*, examined how its population demography is influenced by evolutionary processes, and evaluated the effects of climatic variation over space and time on shaping the current genetic structure.

## Materials and methods

### Population genetics

#### DNA sampling

**Fig 1:**
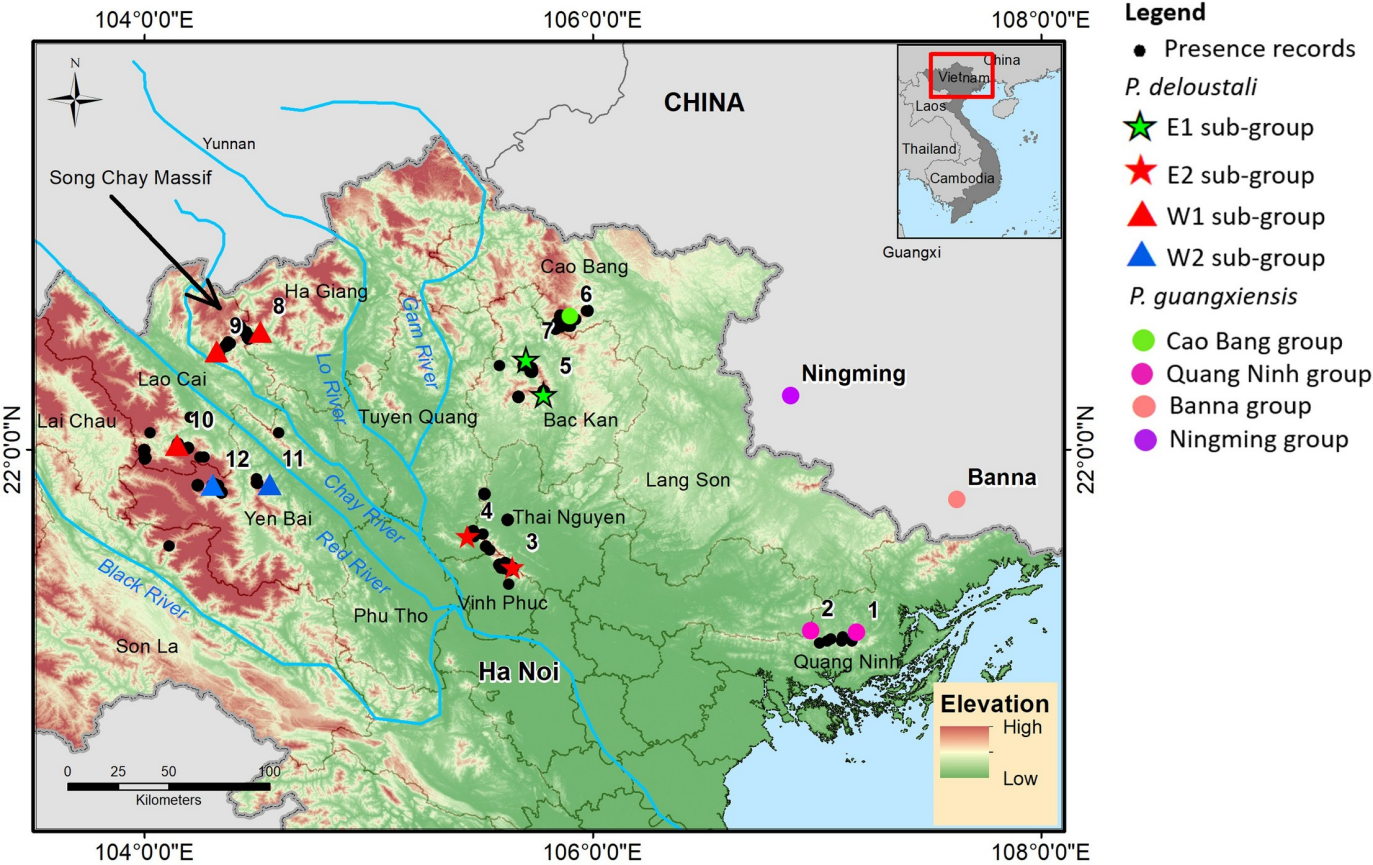
Geographic distribution of samples of warty newts in northern Vietnam. The map was created in ArcMap version 10.2 (ERSI). Blue lines present main rivers. The administrative boundary dataset was downloaded from the GADM (https://gadm.org/download_country_v3.html). The elevation map was extracted from Terrain Elevation Data 2010 (GMTED2010), USGS EROS (http://eros.usgs.gov/#).

In total, 67 muscle samples were taken from the tails of live newts collected at 12 locations during field surveys (Fig 1). The newts were released back into their natural habitat after tissue collection. No animals were euthanized or subjected to anesthesia or analgesia. The tissues were preserved in 99% ethanol in a freezer at Vietnam National University of Forestry, Vietnam. The procedures for collecting specimens and conducting animal operations followed the guidelines for animal experimentation of Kyoto University, Japan (Nos. 20-A-7 and 22-A-2). We extracted total DNA from muscle using the DNeasy Blood & Tissue Kit (Qiagen, Hilden, Germany), following the manufacturer’s protocol. Then the tissues were sequenced for the partial NADH dehydrogenase subunit 2 region (ND2) of mtDNA. The methods used for polymerase chain reaction (PCR), purification of PCR products, and sequencing have previously been described in Nishikawa et al. [40, 41]. We sequenced the amplified fragments using an ABI PRISM 3130 Genetic Analyzer (Applied Biosystems, Carlsbad, CA, USA) at Kyoto University, Japan. A portion of each sample was sent to 1st Base (Malaysia) for sequencing. Then, we assembled the sequences using ChromasPro v.1.34 software (Technelysium Pty Ltd., South Brisbane, Queensland, Australia), and aligned the 1,011 bp ND2 sequences using MAFFT v7.222 (default settings followed a previous study [42]). Then, the sequences were submitted to the DNA Data Bank of Japan (DDBJ; accession number are from LC758635 to LC758702). The ND2 gene has been widely used for genetic studies of newts in the genus *Paramesotriton* [29, 31, 43–48]. In addition, we obtained sequences of *P*. *deloustali* and *P*. *guangxiensis* from GenBank (https://www.ncbi.nlm.nih.gov/), including accession numbers FJ744600 [46], DQ517802, DQ517804 [44], ON794062, ON794063, ON794071, and ON794072 [29]. The sequences listed under accession numbers ON794085 (*P*. *longliensis*), ON794083 (*P*. *caudopunctatus*), ON794119 (*P*. *chinensis*) [31], and KU375031 (*Pachytriton inexpectatus*; https://www.ncbi.nlm.nih.gov/nuccore/KU375031) were used as the outgroup.

#### Phylogenetic and phylogeographic analyses

We used the maximum likelihood (ML) and Bayesian inference (BI) methods to construct phylogenetic trees. To select the best substitution model, we used Kakusan4 [49] to calculate Akaike information criterion (AIC) [50] values for the ML method and Bayesian information criterion [51] values for the BI method. Then, we employed RAxML 8.2 software [52] to produce an ML tree with 1,000 replicates. Nodes were considered to have adequate support when their bootstrap support (BS) values were greater than 70% [53]. We used MrBayes 3.2.6 software [54] with two parallel runs of four Markov chain Monte Carlo (MCMC) methods for 10 million generations to generate the BI tree. Tracer v.1.7 [55] was employed to check the parameter estimates and convergence of BI analysis. Then, we discarded the first 10% of the generations as burn-in. Bayesian posterior probability (BPP) ≥ 95% was the criterion for adequate statistical support of a node. We constructed haplotype networks for all ND2 sequences of *P*. *deloustali* to support the phylogenetic lineages and their geographic structure using the median joining network method in PopArt software [56].

We estimated the divergence times of groups using mtDNA data under a Bayesian molecular clock framework in BEAST v.2.6 [57]. We added two secondary calibration points obtained from previous studies. The first was selected as the node of divergence between the *P*. *caudopunctatus* group and *P*. *chinensis* group of the genus *Paramesotriton*, which has been dated to 23.8 million years ago (Mya; 95% highest posterior density [HPD]: 18.0–29.9 Ma; [48]) as a normal prior (mean 23.8; sigma 3.4; offset 0.0). The second calibration point was placed at the split between *P*. *deloustali* and *P*. *guangxiensis* at 4.96 Mya (95% HPD: 3.55–6.43 Ma; [31]) as a normal prior (mean 4.96, sigma 0.9, offset 0.0). We ran the analysis for 20 million generations, with sampling every 100,000 generations using the HKY85_Gamma function in Kakusan4 [49] with the uncorrelated lognormal relaxed clock model. Then, we used Tracer v.1.7 [55] to assess the stationarity and ensure an effective sample size (ESS) greater than 200 for all estimated parameters. Finally, we employed TreeAnnotator v. 2.6 [57] to generate a maximum group credibility consensus tree with mean node heights, discarding the first 10% as burn-in.

To assess the hypothesis of diversification among groups of *P*. *deloustali* in northern Vietnam, we conducted historical biogeography analysis using parsimony-based statistical dispersal–vicariance analysis (S-DIVA; [58]) in RASP v.3.2 [59]. Based on the natural landscape of northern Vietnam, along with the phylogenetic tree (BI method), haplotype network, and occurrence locations of *P*. *deloustali*, we designated the following four distribution regions: E1, the northeastern area including mountainous area of Tuyen Quang and Bac Kan provinces; E2, the southeastern area including the Tam Dao Mountains in Vinh Phuc, Tuyen Quang, and Thai Nguyen provinces; W1, the northwestern area in Lao Cai and Ha Giang provinces on the right side of the Hoang Lien Son Mountain Range; and W2, the southwestern area covering Yen Bai and Son La provinces, along with the southern area of the Hoang Lien Son Mountain Range.

#### Demographic analysis

To reveal the genetic diversity of the overall population and each major group of *P*. *deloustali*, we calculated haplotype diversity (Hd) and nucleotide diversity (π) using DnaSP v.6 [60]. To assess the demographic history of each group, we performed mismatch distribution analysis by displaying the frequency distribution of pairwise differences [61] between two models: demographic expansion [62] and spatial expansion [63]. The goodness-of-fit of the simulated distribution was evaluated based on expected distributions obtained from a population expansion model. This evaluation was conducted by computing the sum of the square deviation (SSD) with 10,000 replicates. To estimate potential expansion, we conducted neutrality tests for each group using Fu’s *Fs* index [61] with 10,000 simulations. Significant negative values in these tests indicate that a demographic expansion likely occurred in the past. In this study, we implemented mismatch distribution and neutrality tests in Arlequin ver. 3.5 software [64].

To infer the dynamics of effective population size over time for each group, we conducted Bayesian skyline plot (BSP) analysis [65] in BEAST v.2.6 [57]. We used Kakusan4 (Tanabe, 2011) to select the best model (HKY model) for site model input in BEAST. An evolutionary rate of 0.80% divergence per million years for the mitochondrial DNA of Salamandridae [66] was used to construct a strict clock model. Then we ran MCMC analysis with 20 million generations, sampling every 100,000 generations and discarding 10% as burn-in. The parameters were evaluated for convergence using Tracer v.1.7 [55], with an ESS greater than 200. Then the historical demographic events were depicted as a BSP using Tracer v.1.7 [55].

### Species distribution modeling

#### Occurrence data

Presence records of the Vietnam warty newt were gathered from our field surveys conducted from 2018 to 2022 across the entire known geographic range of the species in northern Vietnam. In addition, presence records for this newt were obtained from published documents [67, 68] and from the Global Biodiversity Information Facility (GBIF; https://www.gbif.org/species/2431843). In total, we gathered 146 presence locations of the Vietnam warty newt. To prevent spatial autocorrelation of the occurrence data, we thinned out presence points located within one km of each other by randomly selecting one record and deleting the others; this process was conducted with 10 replicates using the “spThin” package [69]. We used one km as the criterion for thinning presence locations because warty newts have relatively small home ranges. For example, the mean home range of the Hong Kong newt (*Paramesotriton hongkongensis*) was estimated to be only around 0.04 ha [70]. This thinning process reduced the presence locations of Vietnam warty newt to 55 points, which were employed as input data for species distribution modeling (Fig 1).

#### Environmental data

Recent climatic data at the highest available resolution (30 arcsecs) were downloaded from the WorldClim database (http://www.worldclim.com/; [71]). The dataset contains 11 variables of temperature and eight layers of precipitation. To project the paleo-distribution of the Vietnam warty newt, we gathered historical climate datasets representing the mid-Holocene (MH), LGM, and last interglacial (LIG) from the WorldClim database. Climatic data for the MH (~6 thousand years ago [Kya]) and LGM (~21 Kya) were obtained from global climate models of MIROC-ESM [72]. The paleo-climate during the LIG (~140–120 Kya) was reconstructed in a previous study [73]. To prevent autocorrelation among climatic variables, we calculated Pearson’s correlation index (*r*) using ENMTools version 1.4.4 [74] and retained only one variable from each highly correlated pair (|*r*| > 0.80). Ultimately, seven predictors were selected for inclusion in the final models: annual mean temperature (Bio1), mean diurnal range (Bio2), temperature seasonality (Bio4), temperature annual range (Bio7), annual precipitation (Bio12), precipitation in the driest month (Bio14), and precipitation seasonality (Bio15).

#### Ensemble species distribution modeling

The availability of numerous techniques for species distribution modeling, which might show considerable differences in their results, causing difficulty for researchers selecting the most appropriate methodology for their needs [75, 76]. The technique of ensemble species distribution modeling (eSDM) is widely employed for predicting species distributions and functions by averaging the outputs from multiple species distribution models using various algorithms [75, 77–81]. BIOMOD [76, 82, 83] is a highly regarded ensemble package that is widely used for species distribution modeling. In addition, this model is open access, and its usage is expected to persist [79]. Here, we applied the BIOMOD2 package [83, 84] version 4.2–2 of the R programming language [85] to create ensemble models for *P*. *deloustali* with the default settings following the recommendations of Guisan *et al*. [75]. Nine algorithms were run for our models, as suggested by Vaissi [81]: generalized boosted models (GBM, n.trees = 1000, 3-fold cross-validation); random forest (RF, n.trees = 1000); generalized linear models (GLM, type = ‘quadratic,’ interaction.level = 1, stepwise procedure using AIC); generalized additive models (GAM, algo = ‘GAM_mgcv’); multivariate adaptive regression splines (MARS, simple with no interaction); surface range envelope (SRE, quant = 0.025); flexible discriminant analysis (FDA); classification tree analysis (CTA, CV.tree = 50, 5 Fold Cross-Validation); and maximum entropy (MaxEnt.Phillips, https://biodiversityinformatics.amnh.org/open_source/maxent/).

Absence records were not available for the newt, and presence–absence data were required for most of the models (except SRE and MaxEnt). Therefore, pseudo-absence records were randomly created using the BIOMOD_FormatingData function of the BIOMOD2 package [83] with a minimum geographic distance of one km between pseudo-absence points [81, 86] to avoid autocorrelation and cover the distinct environmental conditions across the modeled area [87]. We set the number of pseudo-absence points equal to three times the number of presence points and conducted ten repetitions following the advice of the BIOMOD2 team [84]. For each individual model, we allocated 80% of the data to model calibration and the other 20% to performance assessment of the predictions with four replicates to prevent an effect from splitting the total records [75, 81].

We applied Cohen’s kappa (KAPPA), the area under the receiver operating characteristic (ROC) curve (AUC), and true skill statistic (TSS) to evaluate the performance of each model. KAPPA and TSS values vary from –1 to +1, with a value of +1 indicating perfect performance of the model and values between 0.6 and 0.9 representing excellent model performance [88]. AUC ranges from 0 to +1; AUC scores below 0.6 are considered to indicate poor models, scores between 0.6 and 0.9 indicate moderate models, and scores above 0.9 are classified as excellent models [89]. The mean values of important environmental predictors were calculated and plotted using the BIOMOD2 package version 4.2–2 [84]. In this study, we employed three eSDMs, including a model of the entire species range, as well as West and East group models.

#### Ecological niche comparison

To compare the differentiation between lineages occupying different ecological niches, we used two widely used statistical indices: Schoener’s D index [90] and Hellinger’s I index [91] for identity and background testing. The identity test (also called the equivalency test) is used to determine whether the SDMs of two groups are equivalent or non-equivalent, whereas the background test (also called the similarity test) is generally used to test for niche conservatism or divergence between two groups [91]. The observed niche overlap values between niches of the two groups are compared to the niche overlap values of a null distribution with 100 pseudo-replicates using a one-side test for the identity test (alpha level of 0.05) and a two-sided test for the background test (alpha level of 0.05). For the identity test, if the observed niche overlap value fell within the bottom of 5% of the null distribution, we rejected the hypothesis of niche equivalence. For the background test, observed niche overlap values above the 95% confidence interval of the null distribution suggest that the groups may have similar ecological requirements, which is consistent with the concept of niche conservatism. On the other hand, if observed overlap values are lower than the values expected due to chance, the species may have different ecological requirements, referred to as niche divergence [91]. All of these ecological niche tests were performed using the ENMTools package version 1.0 [92] of the R programming language [85] version 4.2.2.

## Results

### Phylogenetic reconstruction and divergence times

**Fig 2:**
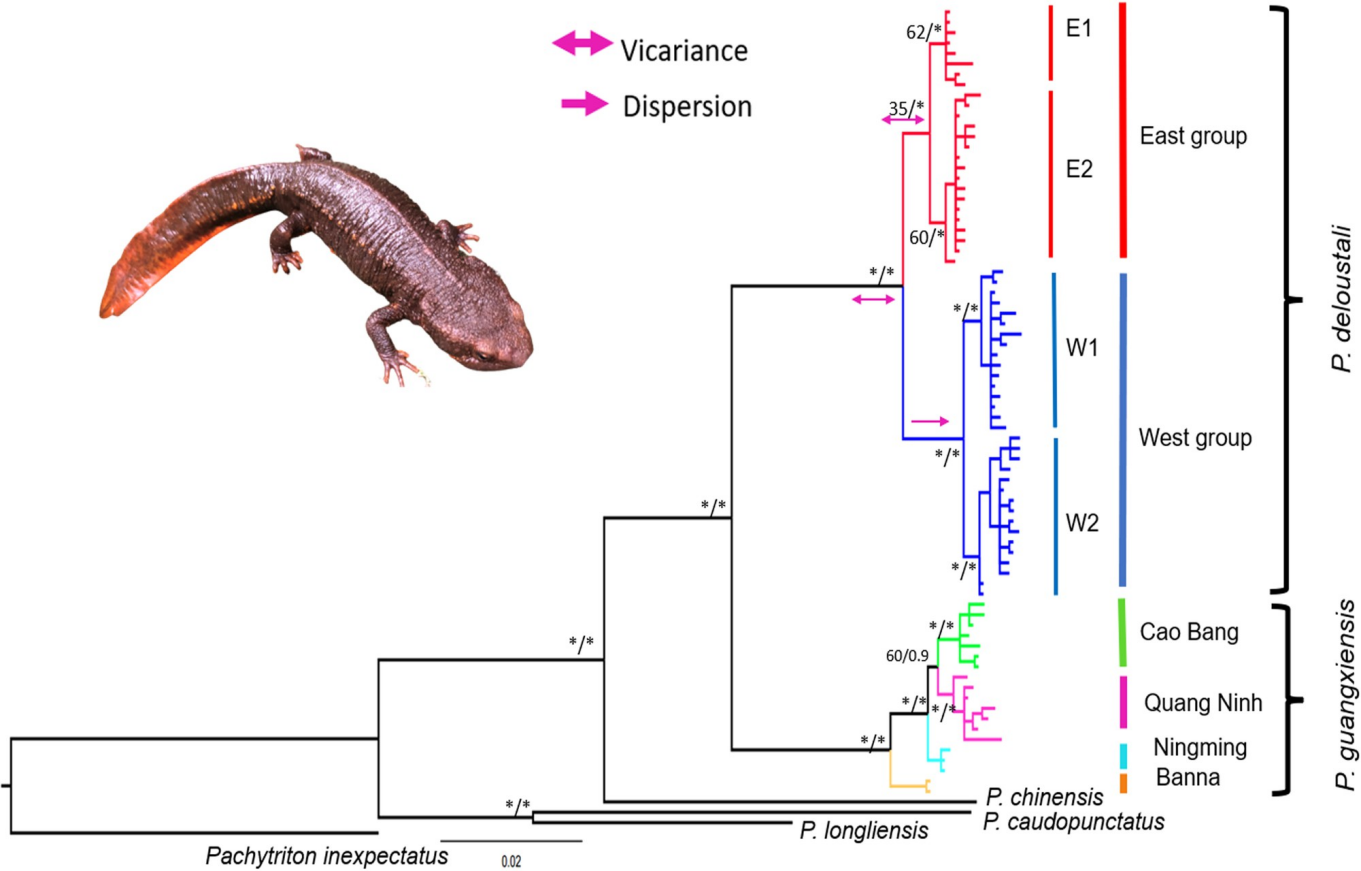
The phylogenetic relationships of warty newts in northern Vietnam based on mitochondrial DNA ND2 sequences. Bootstrap supports (Maximum-Likelihood—ML)/posterior probabilities (Bayesian Inference—BI) were provided for only major nodes. Asterisks indicate adequate statistical support (>70* for ML method, and > 95* for BI method). Pink arrows indicate groups diverging through vicariance/ dispersal (S-DIVA, [58]) performed in RASP 4.3 [59]. Divergence dates between the two main groups of *P*. *deloustali* (East and West) were estimated to be in the Early Pleistocene, approximately 1.92 Mya, with 95% confidence intervals of HPD ranging from 1.25 to 2.75 Mya. The E1 and E2 groups within the East group diverged from a common ancestor at around 0.88 Mya (HPD: 0.45–1.36 Mya). Within the West group, the split between subgroups W1 and W2 occurred at roughly 0.80 Mya (HPD: 0.41–1.21 Mya) (Fig 3).

Phylogenetic reconstruction based on mitochondrial DNA ND2 (1011 bp) revealed two species of warty newts in northern Vietnam (*P*. *deloustali* and *P*. *guangxiensis*). The BI and ML trees are generally identical; therefore, we show only the BI tree in Fig 2. Within *P*. *deloustali*, the BI tree strongly supported two major groups, designated East and West (Fig 2), although the monophyly of the East group was not significantly supported by the ML tree (Fig 2). The East group was distributed across the eastern region of northern Vietnam, on the right banks of the Lo and Gam Rivers (Fig 1). This group could be divided into two subgroups: E1 at locations 5 and 7, and E2 at locations 3 and 4 (Fig 2). The West group occurred in northwestern Vietnam, on the left sides of the Lo and Gam Rivers. This group also included two subgroups: W1 and W2 (Figs 1 and 2). Interestingly, the W1 subgroup covered an area running across the Red River in Lao Cai and Ha Giang Provinces at sampling locations 8, 9, and 10. The W2 group encompassed individuals from locations 11 and 12 in Yen Bai Province (Fig 1).

**Fig 3:**
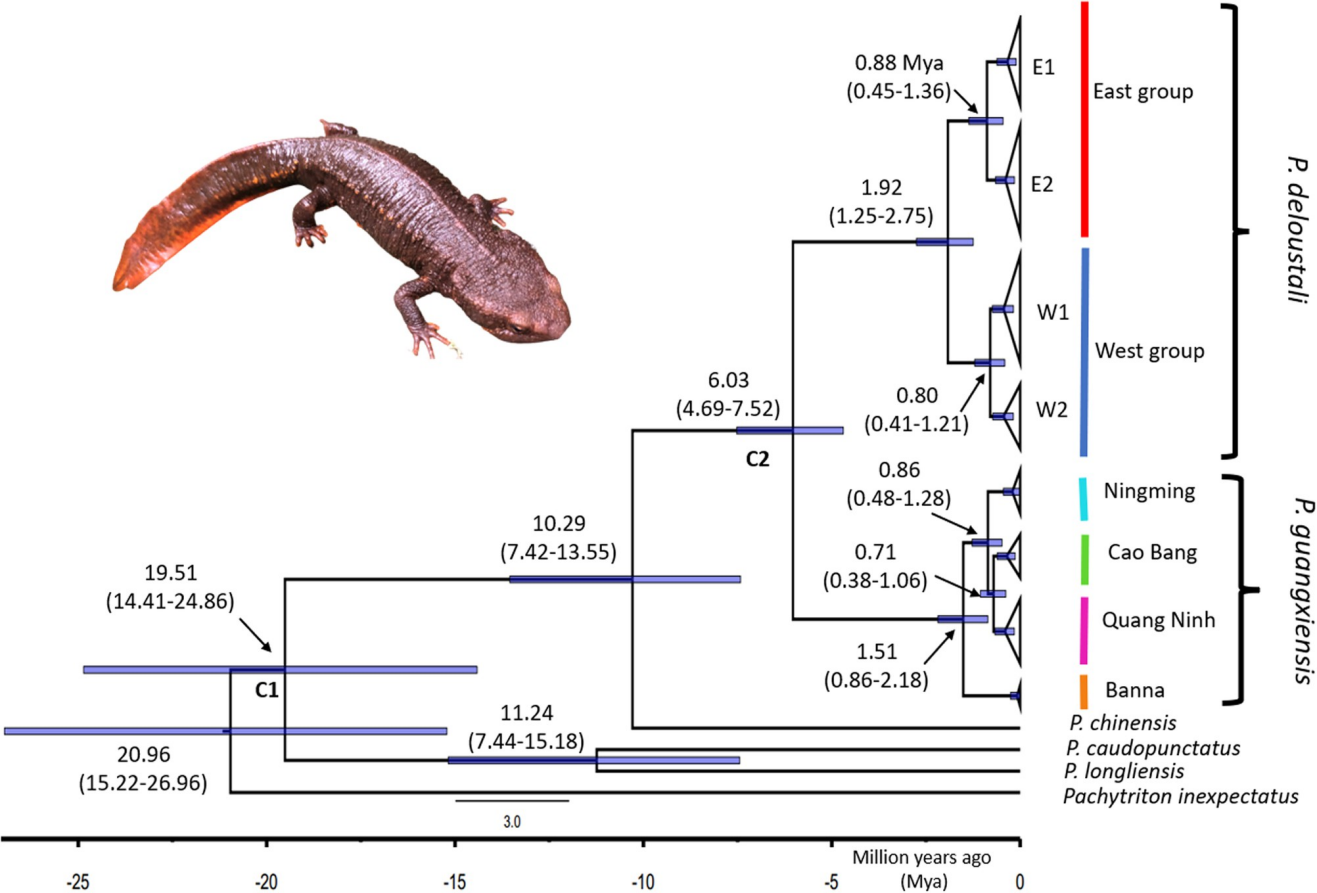
Time-calibrated topology of main populations of newts in northern Vietnam based on mitochondrial DNA ND2. C1 and C2 represent the calibration priors. Blue bar nodes indicate 95% confidence intervals of the highest posterior density (HPD).

Our analysis confirmed the presence of the Guangxi warty newt (*P*. *guangxiensis*) in northern Vietnam at sampling location 6 in Cao Bang Province, in addition to two other populations in Ningming and Banna, China. In particular, our mitochondrial DNA ND2 analysis revealed that the samples from locations 1 and 2 in Quang Ninh Province were *P*. *guangxiensis*, while previous studies have reported this population as *P*. *deloustali* [33] (Figs 1 and 2).

### Phylogeography

**Fig 4:**
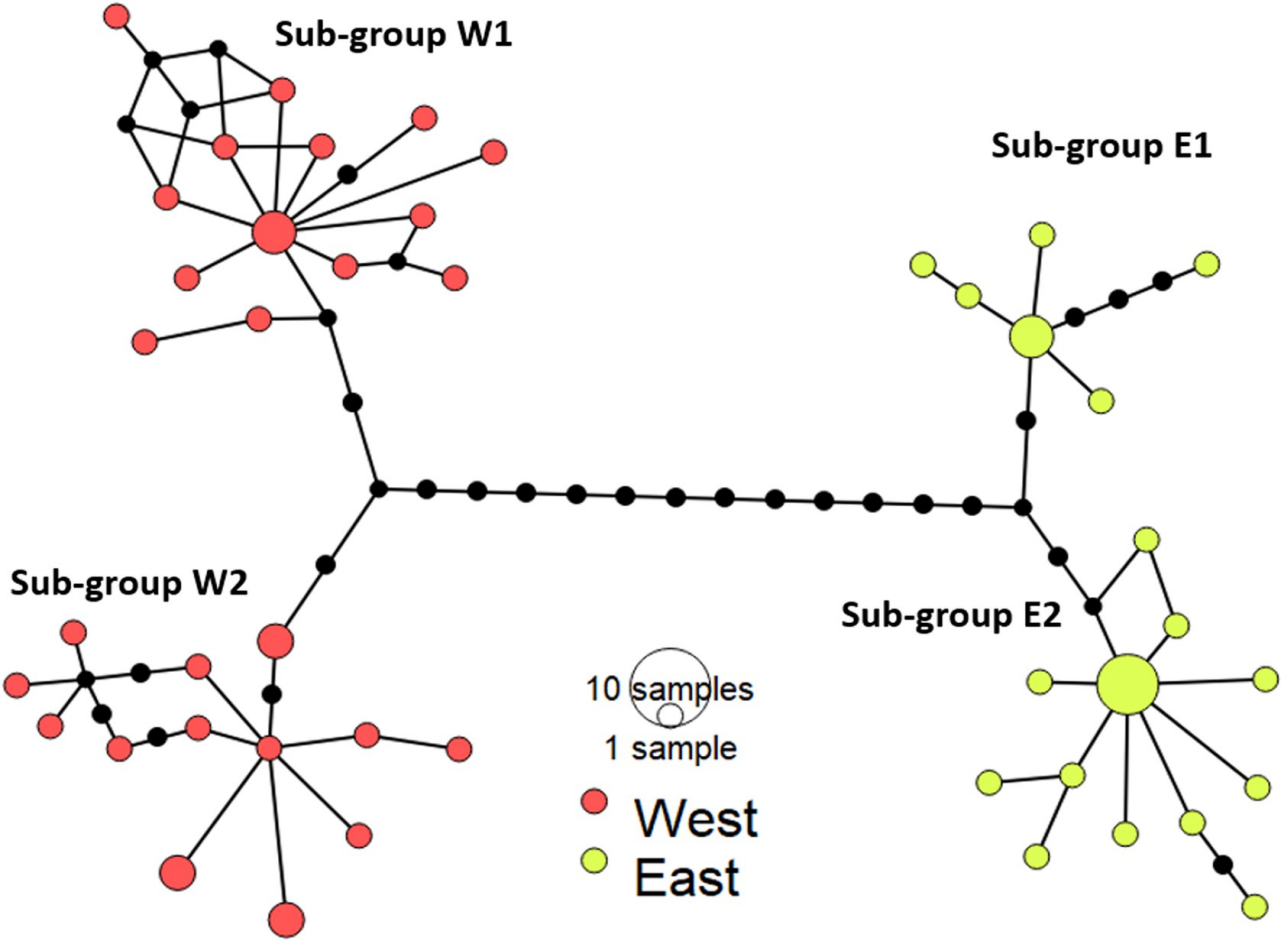
Haplotype network for *P*. *deloustali* based on the mitochondrial DNA ND2 region. Circle diameter presents to the number of individual sharing haplotypes. The color of circles indicates the region of the samples (East and West population). The black dots show the number of mutational steps between individuals.

The numbers of haplotypes in the East and West groups of *P*. *deloustali* were 18 and 27, obtained from 25 and 32 sequences, respectively (Table 1). The haplotype network indicates that the East group was separated from the West group by 13 mutation steps. The subgroups were separated by only four mutation steps (Fig 4).

**Table 1:** Genetic diversity analysis and neutrality tests for each major group of *P*. *deloustali* based on mitochondrial DNA ND2.

Population	N	Nh	Hd ± SD	π ± SD	Fu’s Fs	p-value
Overall	57	45	0.985 ± 0.008	0.01285 ± 0.00033	-23.3626*	<0.001
East	25	18	0.940 ± 0.037	0.00411 ± 0.00051	-10.5066*	<0.001
West	32	27	0.988 ± 0.012	0.00585 ± 0.00032	-20.5022*	<0.001

(Note: N-number of individuals; Nh-number of haplotypes; Hd-haplotype diversity; π-nucleotide diversity; SD-standard devitation. Asterisk indicates significant p-value (<0.01)).

S-DIVA performed for ancestral area reconstruction indicated that the two main groups diverged due to a vicariance event between the east and west (Fig 2 and S1 Fig).

### Historical demography

**Fig 5:**
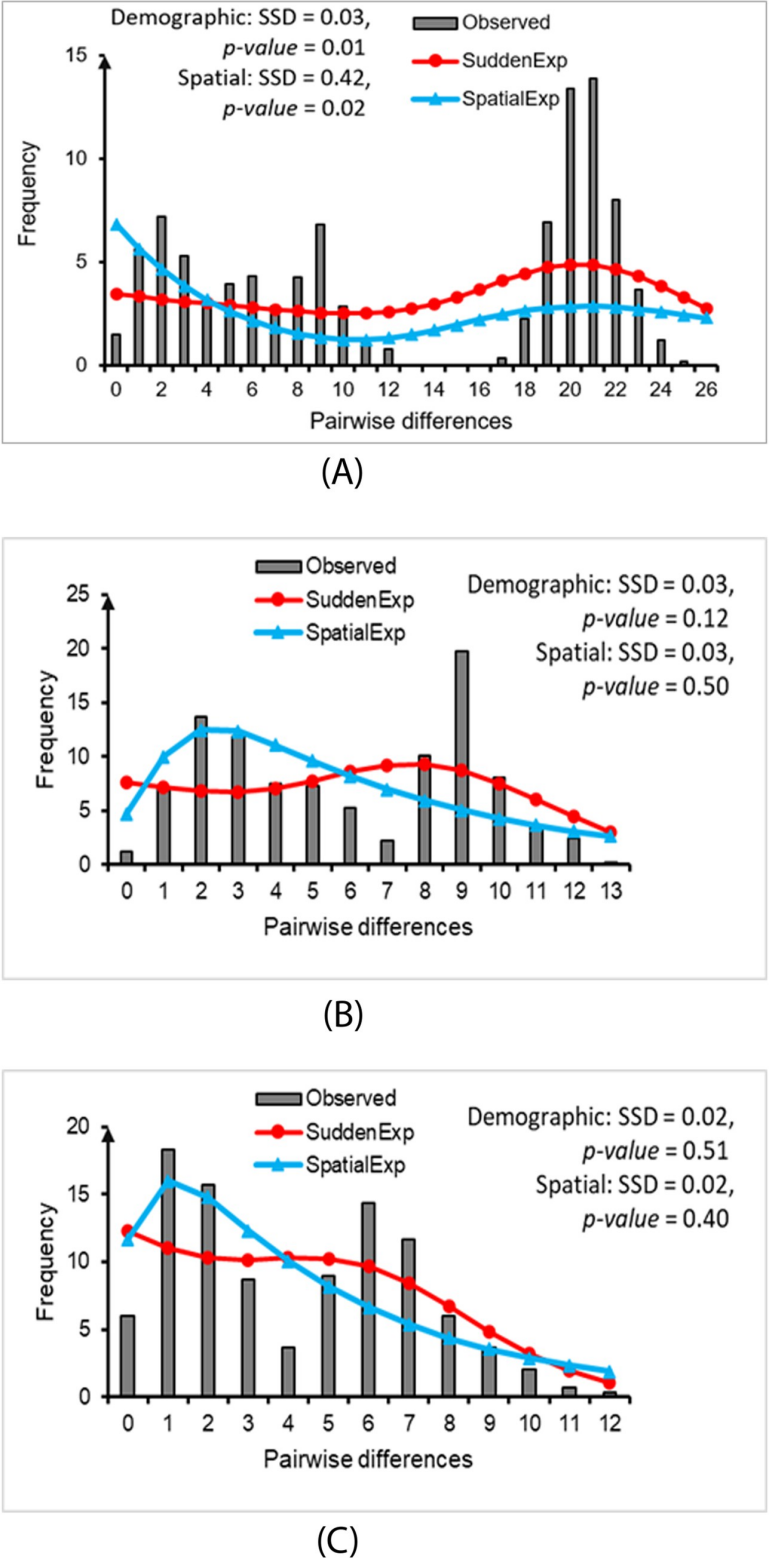
Mismatch distribution analyses based on the mitochondrial DNA ND2 (1011 bp) of *Paramesotriton deloustali*, overall population (A), West group (B), and East group (C). Red and blue lines indicate the demographic expansion, and spatial expansion models, respectively. The sums of squared deviations (SSD) and p-value were presented for each group.

Mismatch distribution analysis revealed multiple peaks for the model of overall population, and two peaks (bimodal) for the models of both the West and East groups (Fig 5), providing evidence that two distinct populations were present within each group [93, 94]. The SSD values for both demographic and spatial expansion were rejected in the analysis of the overall population (p-value < 0.05; Fig 5A). However, they did not support rejecting demographic and spatial expansion for the West and East groups (p-value > 0.05; Fig 5B and 5C). Based on analysis of the sequences of each subgroup, our results indicate a unimodal mismatch distribution for each of the four subgroups (S2 Fig), indicating sudden population expansion events [94, 95]. Based on SSD values, we did not reject the possibility of demographic and spatial expansion for any of the groups (p-value > 0.05; S2 Fig).

Our analysis of genetic diversity revealed high haplotype diversity (Hd) values for the overall population as well as the West and East groups (0.985, 0.940, and 0.988, respectively). However, the nucleotide diversity (π) values were comparatively low, measuring 0.012, 0.004, and 0.005 for the overall population, West group, and East group, respectively (Table 1). Furthermore, the genetic and nucleotide diversity of the West group were higher than corresponding values for the East group. The neutrality test produced significantly negative Fu’s Fs values for all tests (overall population, West, and East group). These results support the proposed pattern of historical demographic expansion for *P*. *deloustali* and each group within this species.

**Fig 6:**
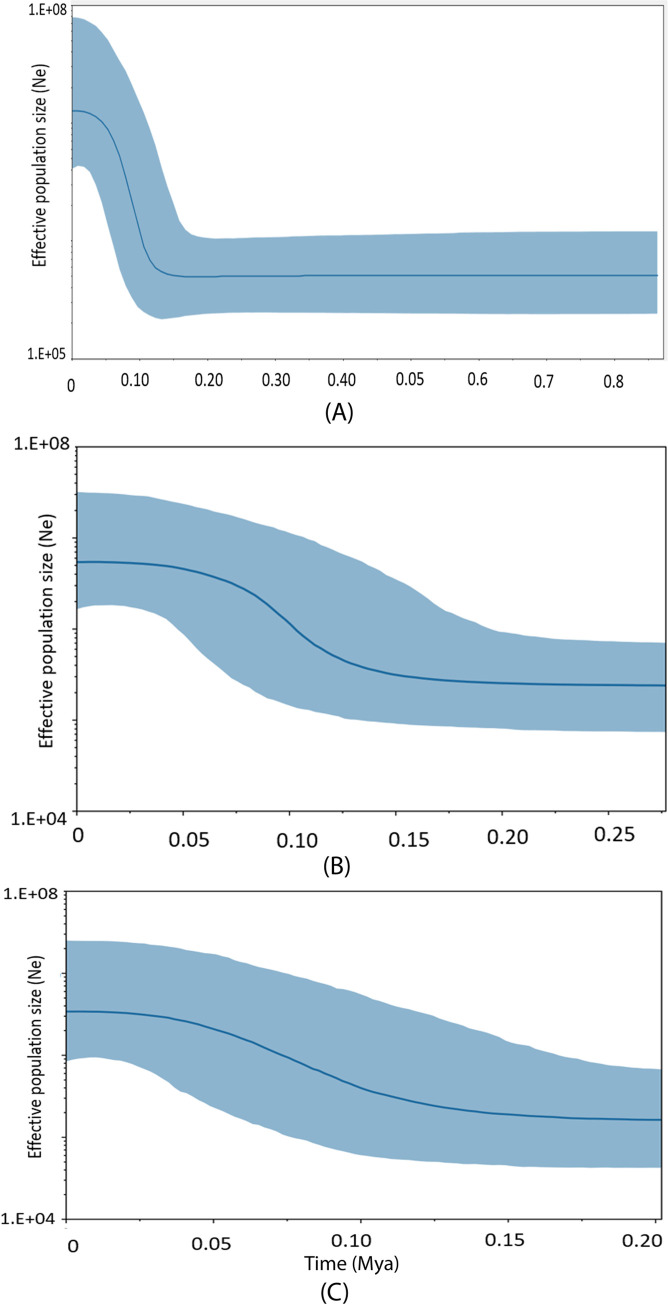
Bayesian Skyline Plots (BSP) presenting demographic variation over time based on mitochondrial DNA ND2 of *Paramesotriton deloustali*: overall population (A) West group (B), East group (C). Dark blue solid lines: median estimates, and light blue area: 95% confidence intervals of highest posterior densities.

Historical demographic estimation using BSPs of the overall population and two main groups of *P*. *deloustali* revealed that a population expansion began around 0.15 Mya. In particular, the effective population size of the West group was estimated to increase sharply from ca. 0.11–0.07 Mya, and then to stabilize from this period to the present (Fig 6B). For the East group, we estimated a steadily increasing effective population size from approximately 0.15–0.04 Mya, which then became stable until the present (Fig 6C).

### Ecological niche models

**Fig 7:**
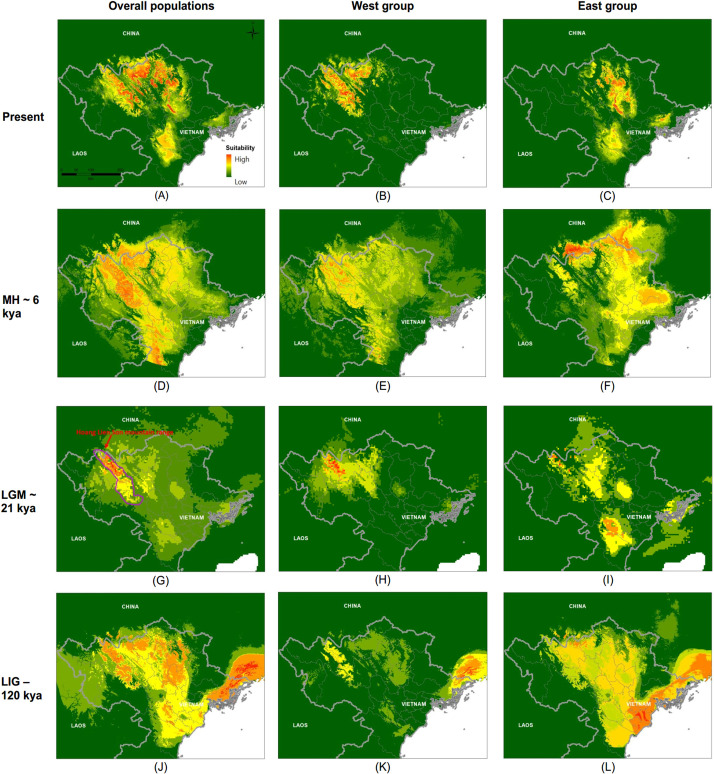
Predicted current distribution and projected historical ranges of overall population, West and East groups of *Paramesotriton deloustali* to the past: Mid Holocene (MH, ~6 Kya), Last Glacial Maximum (LGM, ~21 Kya), Last interglacial (LIG, ~120 Kya). The violet polygon in (G) shows location of Hoang Lien Son Mountain Range.

Ecological niche models of the entire range of *P*. *deloustali* as well as each separate group showed high values of KAPPA, TSS, and AUC, ranging from 0.649 to 1 (S1 Table). This indicates that our models performed better than random prediction. RF was the best-fitting model, whereas SRE showed the worst fitting performance (S1 Table). The most important variable of the model for the entire range species was annual precipitation (Bio12) in five model algorithms (CTA, GBM, MAXENT, RF, SRE), while mean diurnal range (Bio2) made the largest contribution in three models (GAM, GLM, and MARS). For the FDA algorithm, temperature seasonality (Bio4) was the most important predictor (S3 Fig). The potential distribution of *P*. *deloustali* was predicted to cover a wide and fragmented area, including the East and West regions of northern Vietnam, as well as a small area of southern China (Fig 7A). For the West group, annual precipitation (Bio12) was the strongest predictor in all model algorithms (S4 Fig). The predicted suitable area for the group covered two main areas of Yen Bai, Lao Cai, and Ha Giang Provinces and the western part of northern Vietnam (Fig 7B). For the East group, mean diurnal range (Bio2) was predicted as the variable making the largest contribution in all model algorithms, followed by annual precipitation (Bio12; S5 Fig). The estimated range of the East group was mainly continuous, concentrated in the Vinh Phuc, Thai Nguyen, Tuyen Quang, and Bac Kan Provinces in the eastern part of northern Vietnam (Fig 7C).

### Paleodistribution models

Our projected historical distributions of the entire species and major groups of *P*. *deloustali* showed different trends from the LIG (~120 Kya) to the present. For models of the entire species range, the predicted distributions during the MH (~6 Kya) and the LIG represented a much larger area than the current potential distribution, covering most of northern Vietnam (Fig 7D and 7J). However, the high-suitability area in the paleodistribution model for the LGM (~21 Kya) was predicted to be smaller than other models, concentrated in the Hoang Lien Son Mountain Range with an upward shift in elevation, while low-suitability areas covered most of northern Vietnam (Fig 7G). The paleodistribution of the West group was predicted as a small area in the Hoang Lien Son Mountain Range during the LIG and LGM periods (Fig 7K and 7H). During the MH period, the distribution of the West group expanded to cover a larger area, stretching from the east to the west in northern Vietnam (Fig 7E). In the historical distribution models of the East group, a large area ranging from central northern Vietnam to the coast of northern Vietnam and southern China was suitable during the LIG (Fig 7L). The range of this group narrowed and shifted to the west during the LGM (Fig 7I), and covered a significantly larger area during the MH, which was focused in the northeast provinces of Vietnam and southern China (Fig 7F).

### Niche overlap among major groups

**Fig 8:**
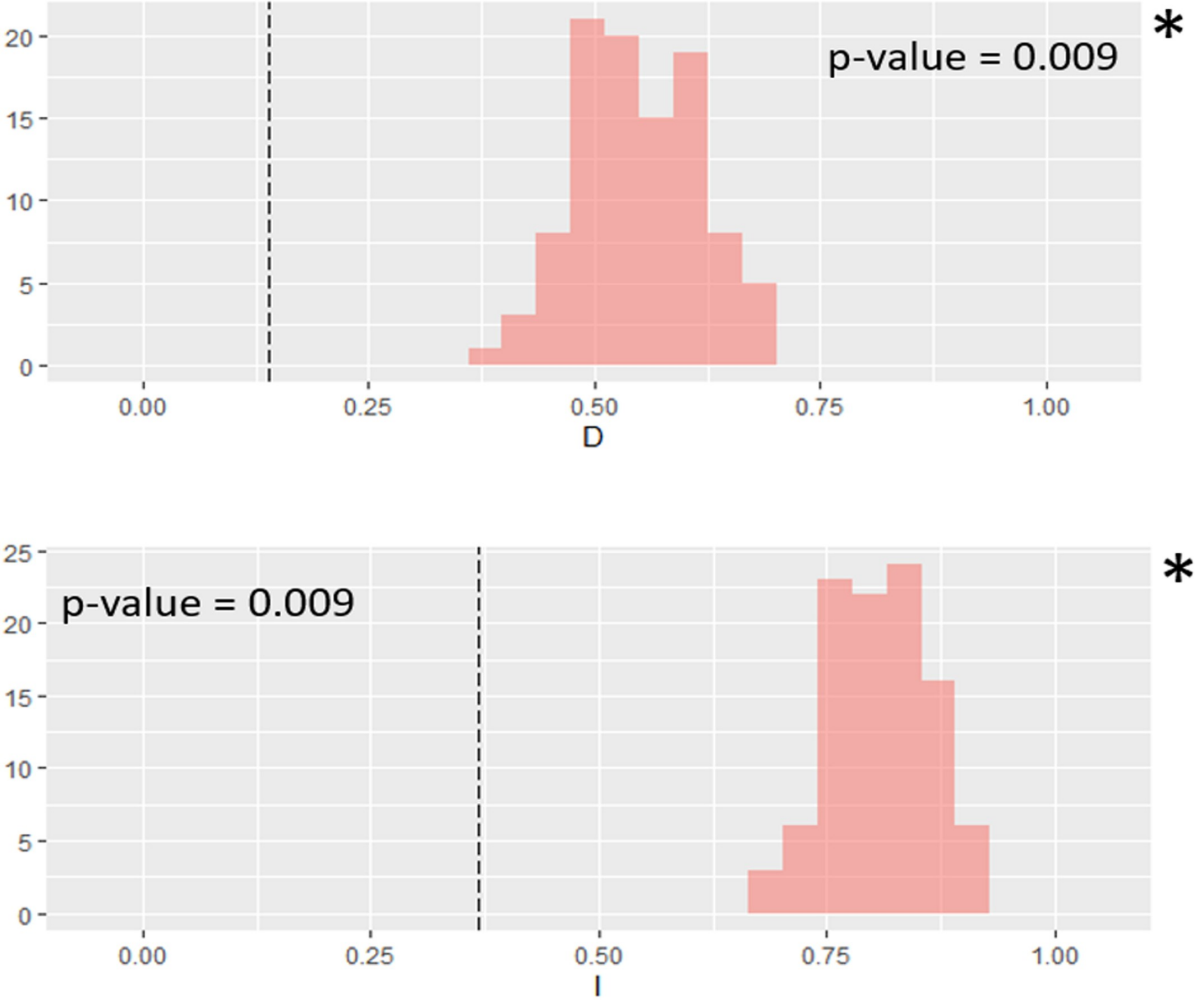
Niche identity test between ecological niche models for groups East and West of *Paramesotriton deloustali*. Asterisk indicates statistical significance (p < 0.05). Dot line presents the true niche overlap values, red column indicates the niche overlap values of the null distribution (100 replicates).

Identity testing between the East and West groups of *P*. *deloustali* showed that the values of Hellinger’s I and Schoener’s D were significant (p-value < 0.05), and the true overlap value was smaller than the overlap of the null distribution (Fig 8), supporting rejection of the null hypothesis in the equivalent test.

**Fig 9:**
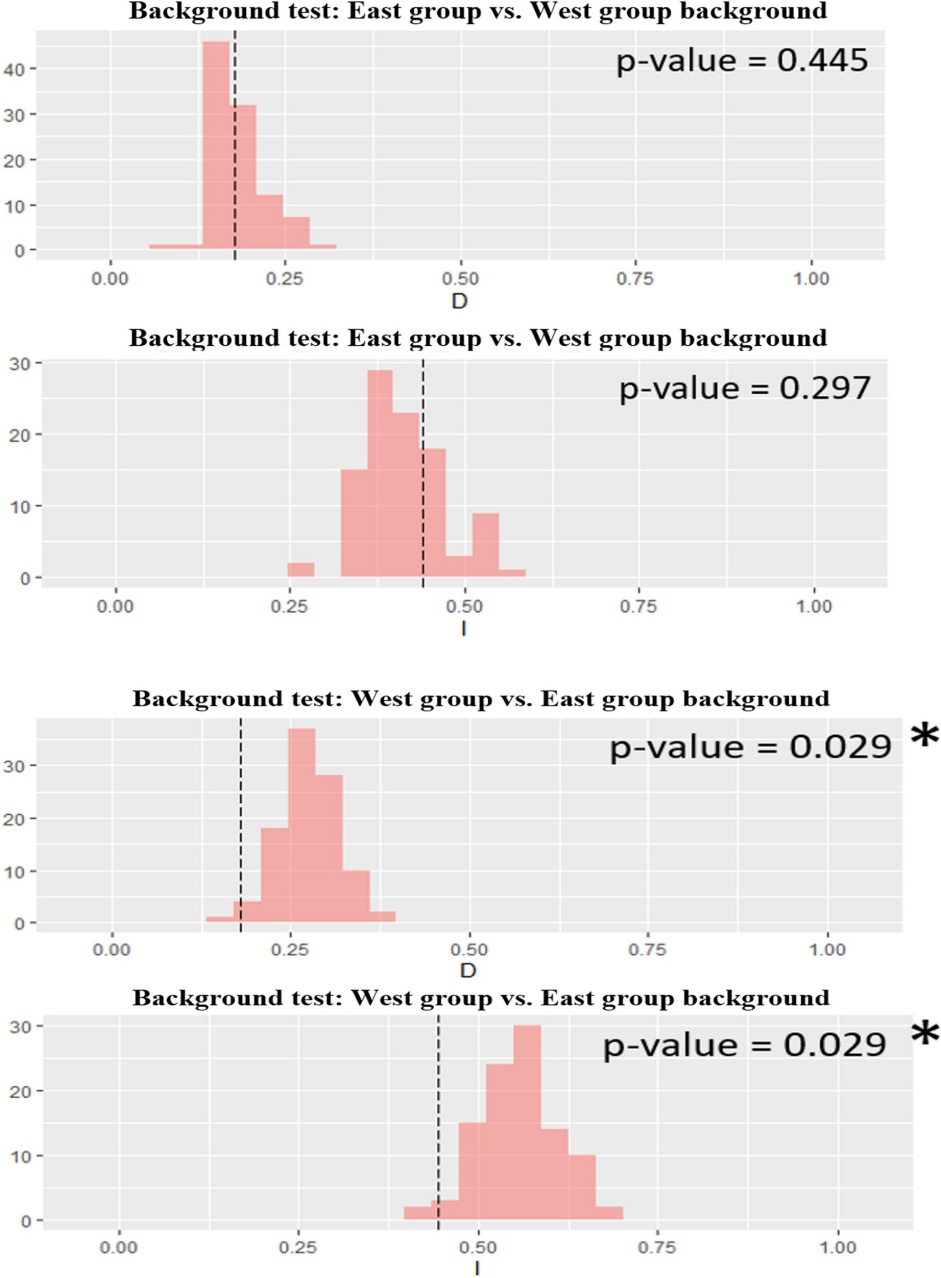
Niche background tests between ecological niche models for groups East and West of *Paramesotriton deloustali*. Asterisk indicates statistical significance (p < 0.05). Dot line presents the true niche overlap values, red column indicates the niche overlap values of the null distribution (100 replicates).

For the background tests, the null hypothesis was not rejected for the direction of the East group versus the background of the West group (p > 0.05 for Hellinger’s-based I; p > 0.05 for Schoener’s D). However, for the direction of the West group versus the background of the East group, the null hypothesis was rejected (p < 0.05 for Hellinger’s-based I; p < 0.05 for Schoener’s D), and the true niche overlap value was lower than the value for the null distribution (Fig 9), indicating niche divergence.

## Discussion

Several studies have attempted to reveal the evolutionary history and geographical distribution of species in the genus *Paramesotriton* based on phylogenetic analysis [28, 43, 44, 48, 96–98] or integration of phylogenetic analysis with ecological niche modeling [29, 31]. However, these studies have primarily focused on genus or species groups. The present study is the first to investigate the influence of historical processes on shaping the genetic structure and distribution within one species, *P*. *deloustali*, at both the species and population levels.

### The boundary of species

*Paramesotriton deloustali* has been observed in several locations in northern Vietnam, including the provinces of Vinh Phuc, Bac Kan, Ha Giang, Yen Bai, Tuyen Quang, Thai Nguyen, Lao Cai, and Quang Ninh [32, 33, 99], as well as Yunnan, China [34]. Our analysis using the BI method strongly supported two major groups (West and East) of *P*. *deloustali* in northern Vietnam. However, the East group was supported with lower confidence based on the ML method (Fig 2). We considered the East and West populations two major geographic groups of this newt species. The same contrast (highly significant in BI but nonsignificant in ML) was found in previous phylogenetic studies of tailed amphibians, including the Shangcheng stout salamander *Pachyhynobius shangchengensis* [100], Black salamander *Aneides flavipunctatus* [101], and other organisms [102, 103]. We generally present the results obtained from BI in the following discussion.

The West group occurs in northwestern Vietnam, covering sampling locations in Yen Bai, Lao Cai, and Ha Giang provinces. By contrast, the East group is distributed across the eastern region of northern Vietnam, encompassing Thai Nguyen, Vinh Phuc, Tuyen Quang and Bac Kan provinces (Fig 1). These results are largely consistent with previous reports, except for the locations in Quang Ninh Province, Vietnam. Nguyen *et al*. [33] identified the newt population in Quang Ninh as *P*. *deloustali*. However, our analysis of ND2 mitochondrial DNA revealed that this population was nested within *P*. *guangxiensis* (Fig 2). Furthermore, the analysis of morphological characteristics revealed a significant difference between the newts in Quang Ninh Province and other newt populations (Dung Tran 2023, unpublished data). We also highly recommended the use of nuclear DNA analysis in confirming the taxonomy classification of newt in the Quang Ninh province.

Recently, *P*. *deloustali* has been listed as “Least Concern” on the IUCN Red List due to its widespread distribution [32]. However, our findings suggest that the population in Quang Ninh may not be *P*. *deloustali*, which would greatly reduce the geographic distribution of this species. The distribution map on the IUCN’s official website currently shows a broad range for this species, including areas of Quang Ninh Province [32]. We highly recommend revision of the current distribution map to accurately represent the range of this species, which will provide useful data for conservationists and wildlife managers undertaking conservation activities (e.g., conducting field surveys, long-term monitoring, assessing threats, establishing and managing protected areas) [104]. *Paramesotriton deloustali* is rarely found in lowlands, and most of our observed populations are associated with cool streams in mountainous areas (Dung Tran, personal observations). Increasing areas of agricultural lands, as well as water pollution and flooding in lowland areas, have degraded its habitat [32, 35], likely contributing to the current isolation of populations in high mountain regions. Based on our analysis of genetic structure and ecological niche, we strongly recommend prioritizing conservation efforts for the species in mountainous areas located in Vinh Phuc, Thai Nguyen Province (Tam Dao National Park), Tuyen Quang Province (Ba Be National Park), Yen Bai, and Lao Cai Province. These regions have been identified as having high suitability for the species, but they also exhibit significant isolation. Thus, focusing on conservation initiatives in these areas would be crucial for the long-term preservation of the *P*. *deloustali*. Our models were assembled from nine different species distribution model algorithms but considered the influence of climatic predictors only at a large scale, providing insights related to macroecology, biogeography, and conservation biology [105, 106]. Future studies using finer-scale models with more presence locations, specific vegetation layers, and human footprint variables should be conducted to increase the accuracy of model results for practical conservation purposes. Furthermore, our genetic data and eSDM results indicate that the populations of *P*. *deloustali* exhibit low nucleotide diversity and are generally isolated in fragmented areas. This isolation may reduce the effective population size of the species. Thus, conservation efforts for *P*. *deloustali* should prioritize the preservation of all populations.

### Diversification and ecological niche differentiation

The presence of two major groups of *P*. *deloustali* was strongly supported based on the BI tree, haplotype network, and eSDM analysis (Figs 2, 4 and 7). Ecological niche comparison showed significant differences between the ecological niches of the West and East groups. As expected, the identity test revealed that ecological niches were non-equivalent, demonstrating a lack of capacity for ecological exchange between the populations. Background tests showed significance in the direction of the West group against the background of the East group, but not the opposite (Fig 9). According to Warren *et al*. [91], background test results with significance in one direction based on the null hypothesis indicate that the groups may be more similar than expected. Therefore, we considered that these two groups show evidence of ecological niche divergence, indicating that their ecological niches are less similar than would be expected under the null model. Despite both groups inhabiting cool montane streams (Dung Tran, personal observation; [35]), their niches showed evidence of divergence. This was likely due to the niches of these species having different environmental conditions in their respective allopatric ranges, despite their similar niche preferences [31]. In addition, the higher haplotype and nucleotide diversity of the West group may allow it to adapt better to climatic and elevational variation than the East group. Our ecological tests showed a difference in niches of *P*. *deloustali* at the population level, supporting a west-east distribution pattern. This finding is consistent with the hypothesis of west-east evolutionary divergence within the genus *Paramesotriton* [28, 29, 31] as well as other amphibians and reptiles in southern China [107–109].

The time of divergence between two major groups of *P*. *deloustali* was estimated at around 1.92 Mya (HPD: 1.25–2.75 Mya), in the early Pleistocene epoch and Quaternary period (Fig 3), consistent with the estimate of 1.86 Mya (HPD: 0.92–3.06 Mya) reported by Lou *et al*. [29]. The combined effects of geology and climate change likely triggered the diversification of several species of *Paramesotriton* [28, 29]. Northern Vietnam is characterized by abundant karst formations dating to the mid-Devonian through early Triassic periods and the Hoang Lien Son Mountain Range, which uplifted around 65 Mya [16]. Dramatic increases in the strength of monsoons and droughts in Indochina and southern China have occurred several times [110]. The increase event during the late Pliocene and Pleistocene epochs [110] was consistent with the divergence date of the two major groups of *P*. *deloustali* (Fig 3). Therefore, historical climate change likely played a more significant role in promoting the divergence of the East and West groups than geological processes.

**Fig 10:**
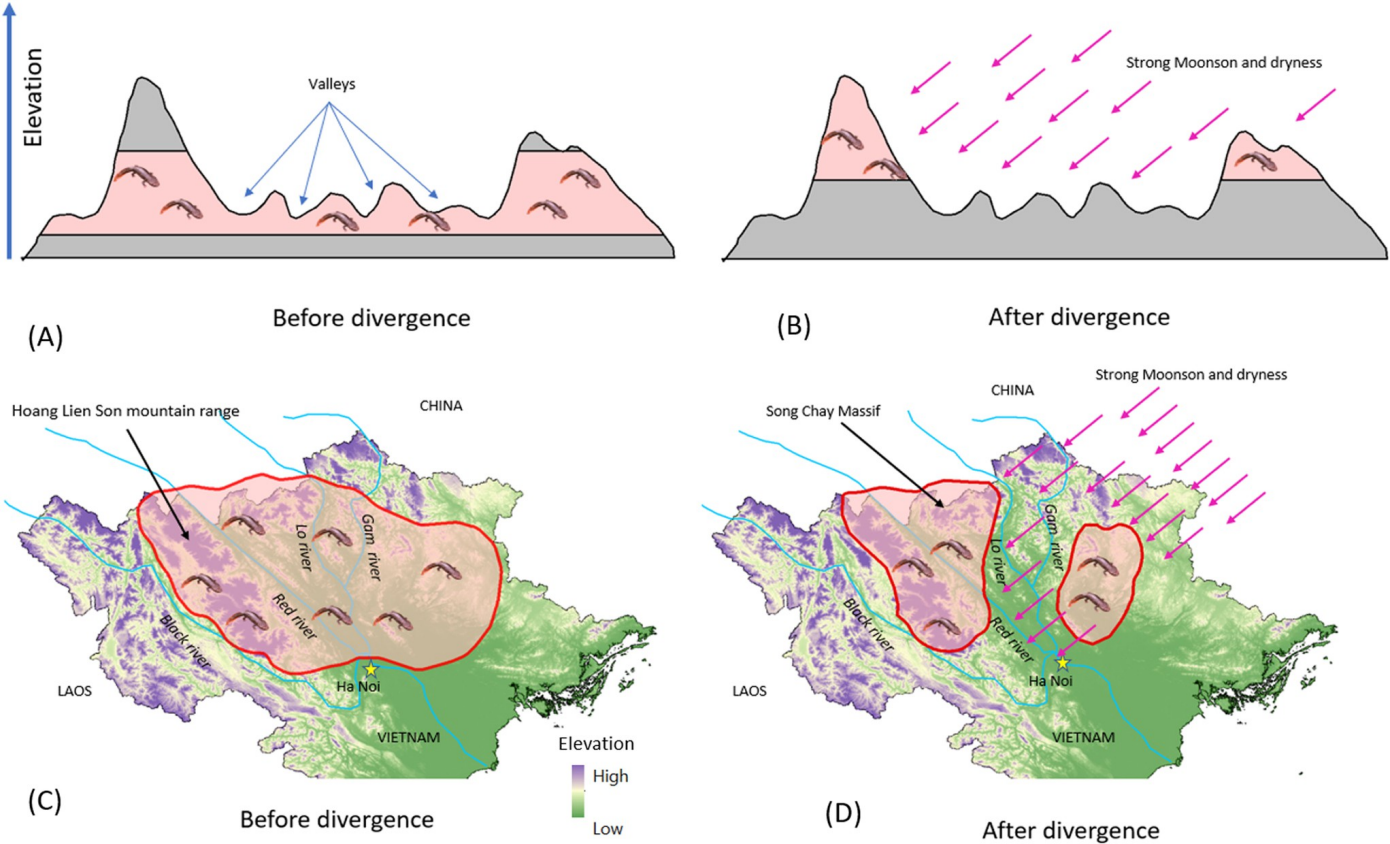
Illustration on the divergence of two main groups of *P*. *deloustali* in northern Vietnam under the effects of historical climate change. The administrative boundary dataset was downloaded from the GADM (https://gadm.org/download_country_v3.html). The elevation map was extracted from Terrain Elevation Data 2010 (GMTED2010), USGS EROS (http://eros.usgs.gov/#).

Yuan *et al*. [31] indicated that several valleys among the mountains served as major dispersal obstacles, driving the diversification of warty newts in south China. For *P*. *deloustali*, wide valleys in the basins of the Lo River and Gam River on the upper side (Tuyen Quang Province) and the Red River on the lower side (Phu Tho and Vinh Phuc Provinces) of the range are potential dispersal barriers for the East and West groups during periods of historical climate change. These barriers prevented newt populations from spreading throughout the lowland valleys and isolated them in cool streams among high mountains (Fig 10). The results of S-DIVA analysis based on biogeographical history strongly supported that the current distributions of the East and West groups were shaped by vicariance events (Fig 2).

The Red River in northern Vietnam serves as a major boundary in the transition zone between subtropical and tropical climates [111]. This river has also been considered a natural barrier for many organisms [36, 112–115]. However, our genetic analysis of *P*. *deloustali* showed that the West group occurred on both sides along the upper part of the river at locations 8, 9, and 10 in Ha Giang and Lao Cai provinces (Fig 1). The northeast region of this area is shielded by a large domal structure with an elevation greater than 2000 m asl, known as the Song Chay Massif (Fig 10), which formed during the Proterozoic era between 2500 and 538 Mya [116]. It is likely that this massif dampened the effect of monsoons in the area during the Pleistocene period, thereby preventing divergence of the newt population. In addition, the valleys formed by the Red River and Chay River are relatively narrow in that area (Fig 1), possibly supporting migration between the two sides of the river by aquatic amphibians such as *P*. *deloustali*.

### Historical demographic and distribution dynamics

Mismatch distribution analysis and neutrality testing provided evidence of historical demographic and spatial expansions of *P*. *deloustali* (Figs 5, S2 Fig, Table 1). In addition, the BSP illustrated that the species began its effective population size expansion around 0.15–0.11 Mya (Fig 6), aligning with the period of the LIG (0.14–0.12 Mya; [73]). Our eSDM analysis predicted expansion of the paleodistribution of *P*. *deloustali* during the LIG (Fig 8). Despite the absence of glaciers, southern China and Indochina experienced relatively low temperatures throughout the LIG [17, 18, 117]. This cooler climate likely promoted expansion of both the population and geographic range of *P*. *deloustali*, a cold-adapted newt [35]. However, the predicted distribution of the West group showed contraction, whereas the range of East group expanded during the LIG (Fig 7K and 7L). In northern Vietnam, western areas have numerous high mountain ranges, particularly the Hoang Lien Son Mountain Range, that are absent from the east area [16]. High mountains can serve as barriers that hinder the movement of cold air masses [118, 119]. Therefore, the presence of high mountainous topography likely reduced the influence of cold air during the early stages of the LIG period, decelerating expansion of the West group. This delay in expansion was supported by the results of the BSP: demographic expansion of the West group began at approximately 0.11 Mya, whereas the East group’s expansion commenced around 0.15 Mya (Fig 6B and 6C).

Our demographic reconstruction results also revealed that *P*. *deloustali* maintained stable populations from approximately 0.7–0.4 Mya to the present (Fig 6). No signs of bottleneck events were found in the estimation of effective population size; however, the eSDM results indicate a reduction in the suitable range during the LGM in models for the overall population as well as the East and West groups (Fig 7). The contrasting results of demographic and species distribution modeling suggest a high population density of this species at the LGM [120]. Interestingly, suitable habitat areas tended to narrow down to high mountain ranges, such as the Hoang Lien Son Mountain Range (Fig 8G). During the LGM, the temperature in Indochina might have decreased by 6–7°C, and precipitation was approximately 30–50% lower than today [121]. The area of tropical rain forests probably decreased, being replaced by wetlands and grasslands in low-lying areas [17, 19, 121] but was maintained in mountainous areas [122, 123]. As a species requiring tropical montane forest for survival, *P*. *deloustali* was likely forced to relocate to high-elevation regions where rainforest persisted during the LGM. Refugia, which are areas with stable climates since the LGM, provide consistent habitats for endemic fauna [124]. Maintenance of suitable habitat and the stable effective population size of *P*. *deloustali* during the LGM indicate that mountainous areas (e.g., the Hoang Lien Son Mountain Range) in the western portion of northern Vietnam are potential refugia over that period of climate change. The Hoang Lien Son Mountain Range is considered to be an important site for biodiversity conservation due to its high level of endemism [125, 126]. Information on historical refugia can improve our understanding of ecological resilience and migration patterns in response to changing climates, providing insights into how populations may respond to future climate changes [127].

We found that the suitable distribution of *P*. *deloustali* increased significantly and expanded to lowland areas during the MH (~6 Kya) for all models of the overall population and the East and West groups (Fig 7). Previous studies have indicated the recovery of lowland rainforest in this region after the LGM [128, 129]. Furthermore, Li *et al*. [129] presented evidence based on pollen deposition that northern Vietnam likely experienced a cool climate during the period of 6.5–5.2 Kya, which would promote the expansion of populations inhabiting high mountains into lowland areas. This possibility is consistent with the hypothesis of elevation shift: cold-adapted taxa occupying high-elevation regions expand into lower-elevation regions during periods of cool climate [130]. The same expansion trend has been found in several studies of tropical or subtropical amphibians, including the Santa Barbara treefrogs *Bokermannohyla alvarengai* and *Bokermannohyla oxente* [131].

In this study, we documented the demographic and distribution dynamics of a montane warty newt driven by historical climate changes. Changes in the historical climate promoted the divergence of two major populations of the newt, and significantly impacted its distributions during the LIG, LGM, and MH, as well as at present. In addition, our eSDM model revealed that both temperature and precipitation variables (mean diurnal range [Bio2] and annual precipitation [Bio12]) play important roles in shaping the distribution of *P*. *deloustali*. Therefore, we predict that this species could be considerably impacted by the future effects of climate change.

## Supporting information

S1 Fig Reconstructed ancestral area of *Paramesotriton deloustali* using Statistical Dispersal-Vicariance Analysis (S-DIVA, Yu et al. 2010) performed in RASP 4.3 (Yu et al.,2015).
(DOCX)

S2 Fig Mismatch distribution of four sub-groups of *Paramesotriton deloustali*. (DOCX)

S3 Fig The contribution of environmental variables in Generalized Boosted Models (GBM), Random Forest (RF), Generalized Linear Models (GLM), Generalized Additive Models (GAM), Multivariate Adaptive Regression splines (MARS); Surface Range Envelops (SRE); Flexible Discriminant Analysis (FDA), Classification Tree Analysis (CTA) and Maximum Entropy (MaxEnt) models projecting for the Vietnam warty newt (*Paramesotriton deloustali*) for entire species range model. (DOCX)

S4 Fig The contribution of environmental variables in Generalized Boosted Models (GBM), Random Forest (RF), Generalized Linear Models (GLM), Generalized Additive Models (GAM), Multivariate Adaptive Regression splines (MARS); Surface Range Envelops (SRE); Flexible Discriminant Analysis (FDA), Classification Tree Analysis (CTA) and Maximum Entropy (MaxEnt) models projecting for the Vietnam warty newt (*Paramesotriton deloustali*) of the West group model. (DOCX)

S5 Fig The contribution of environmental variables in Generalized Boosted Models (GBM), Random Forest (RF), Generalized Linear Models (GLM), Generalized Additive Models (GAM), Multivariate Adaptive Regression splines (MARS); Surface Range Envelops (SRE); Flexible Discriminant Analysis (FDA), Classification Tree Analysis (CTA) and Maximum Entropy (MaxEnt) models projecting for the Vietnam warty newt (*Paramesotriton deloustali*) of the East group model. (DOCX)

S1 Table The value of Cohen’s kappa (KAPPA), ROC curve (AUC), and True skill statistic (TSS) of Generalized Boosted Models (GBM), Random Forest (RF), Generalized Linear Models (GLM), Generalized Additive Models (GAM), Multivariate Adaptive Regression splines (MARS); Surface Range Envelops (SRE); Flexible Discriminant Analysis (FDA), Classification Tree Analysis (CTA) and Maximum Entropy (MaxEnt) models projecting for the Vietnam warty newt (*Paramesotriton deloustali*) for entire range species, East group and West group. (DOCX)
