# Supplementary material for: Demographic and ecological niche dynamics of the Vietnam warty newt, Paramesotriton deloustali: Historical climate influences
Source: PLoS One. 2023 Aug 18;18(8):e0290044. doi: 10.1371/journal.pone.0290044 (PMC10437943; doi:10.1371/journal.pone.0290044)

**S1 Fig. Reconstructed ancestral area of *Paramesotriton deloustali* using Statistical Dispersal-Vicariance Analysis (S-DIVA, Yu et al., 2010) performed in RASP 4.3 (Yu et al., 2015).**


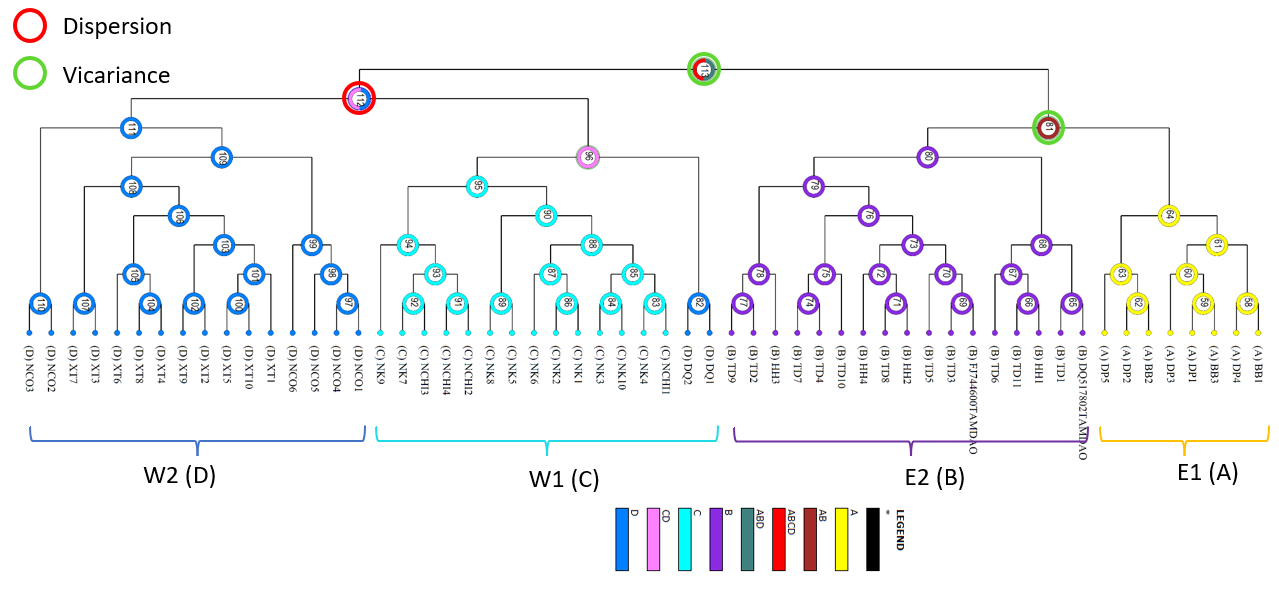

Supplement: S1 Fig — 2010) performed in RASP 4.3 (Yu et al.,2015). (DOCX) [file pone.0290044.s001.docx]
