# Supplementary material for: Demographic and ecological niche dynamics of the Vietnam warty newt, Paramesotriton deloustali: Historical climate influences
Source: PLoS One. 2023 Aug 18;18(8):e0290044. doi: 10.1371/journal.pone.0290044 (PMC10437943; doi:10.1371/journal.pone.0290044)
Supplement: S2 Fig — (DOCX) [file pone.0290044.s002.docx]

**S2 Fig. Mismatch distribution of four sub-groups of *Paramesotriton deloustali***

| **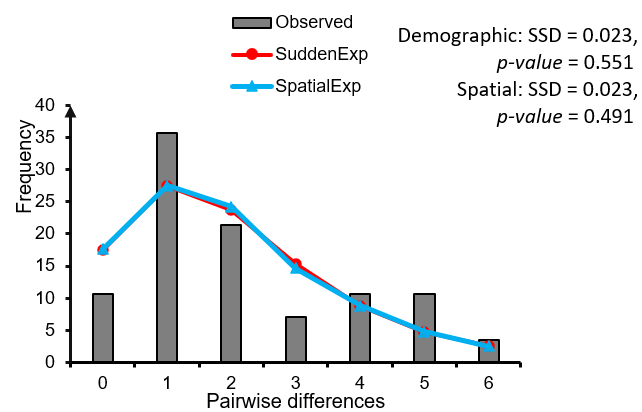**  **Sub-group E1 (East 1)** | **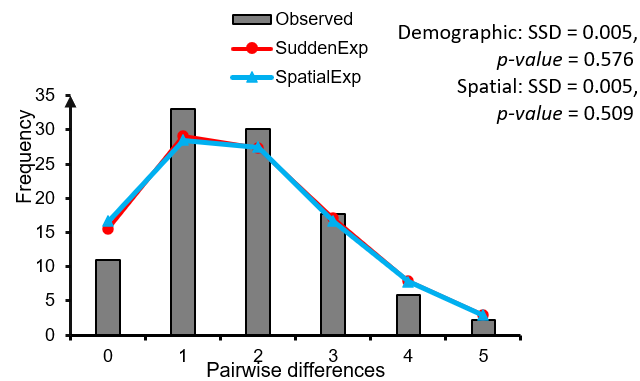**  **Sub-group E2 (East 2)** |
| --- | --- |
| **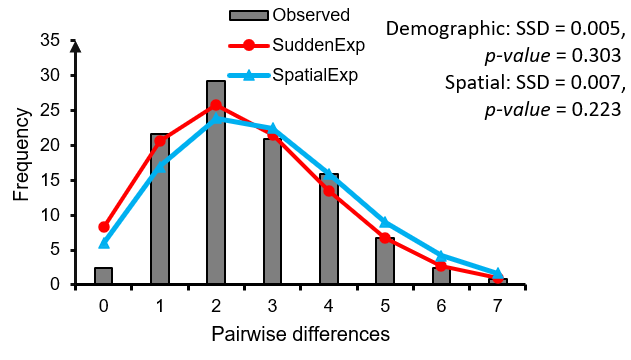**  **Sub-group W1** | 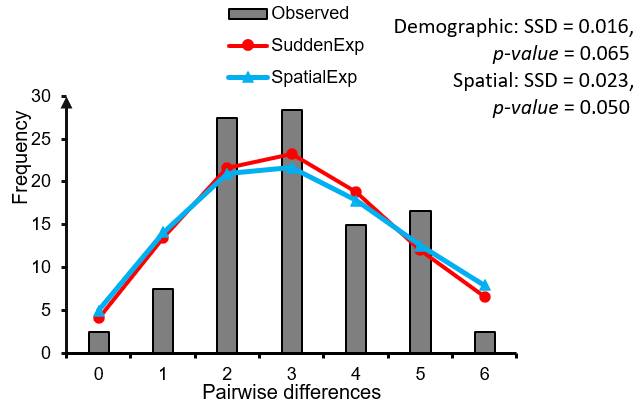  **Sub-group W2** |
