# Supplementary material for: Demographic and ecological niche dynamics of the Vietnam warty newt, Paramesotriton deloustali: Historical climate influences
Source: PLoS One. 2023 Aug 18;18(8):e0290044. doi: 10.1371/journal.pone.0290044 (PMC10437943; doi:10.1371/journal.pone.0290044)

**S4 Fig. The contribution of environmental variables in** Generalized Boosted Models (GBM), Random Forest (RF), Generalized Linear Models (GLM), Generalized Additive Models (GAM), Multivariate Adaptive Regression splines (MARS); Surface Range Envelops (SRE); Flexible Discriminant Analysis (FDA), Classification Tree Analysis (CTA) and Maximum Entropy (MaxEnt) models projecting for the Vietnam warty newt (*Paramesotriton deloustali*) of the West group model.


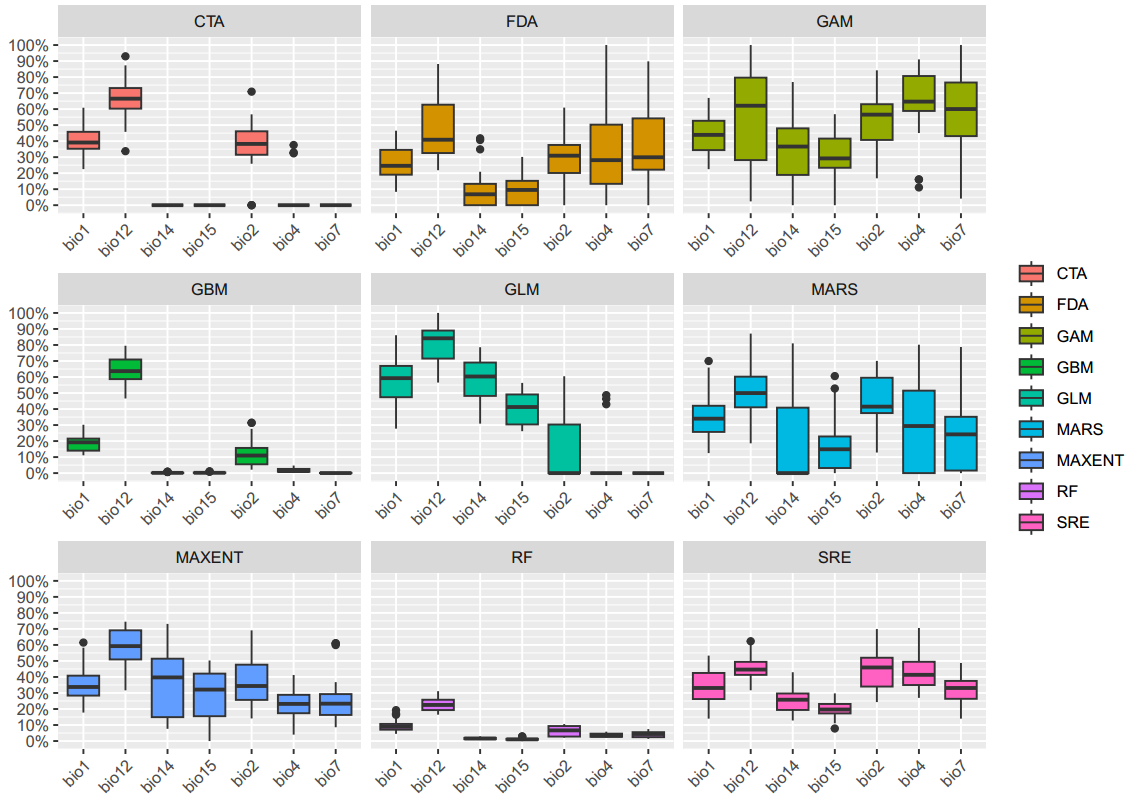

Supplement: S4 Fig — (DOCX) [file pone.0290044.s004.docx]
