# Supplementary material for: Demographic and ecological niche dynamics of the Vietnam warty newt, Paramesotriton deloustali: Historical climate influences
Source: PLoS One. 2023 Aug 18;18(8):e0290044. doi: 10.1371/journal.pone.0290044 (PMC10437943; doi:10.1371/journal.pone.0290044)
Supplement: S1 Table — (DOCX) [file pone.0290044.s006.docx]

**S1 Table.** The value of Cohen’s kappa (KAPPA), ROC curve (AUC), and True skill statistic (TSS) of Generalized Boosted Models (GBM), Random Forest (RF), Generalized Linear Models (GLM), Generalized Additive Models (GAM), Multivariate Adaptive Regression splines (MARS); Surface Range Envelops (SRE); Flexible Discriminant Analysis (FDA), Classification Tree Analysis (CTA) and Maximum Entropy (MaxEnt) models projecting for the Vietnam warty newt (*Paramesotriton deloustali*) for entire range species, East group and West group.

| **Model** | | **GBM** | **RF** | **GLM** | **GAM** | **MARS** | **SRE** | **FDA** | **CTA** | **MaxEnt** |
| --- | --- | --- | --- | --- | --- | --- | --- | --- | --- | --- |
| **Entire species** | **KAPPA** | 0.899±0.018 | 0.973±0.015 | 0.924±0.052 | 0.928±0.054 | 0.888±0.027 | 0.687±0.026 | 0.850±0.026 | 0.816±0.067 | 0.865±0.063 |
|  | **AUC** | 0.992±0.002 | 0.999±0.001 | 0.989±0.009 | 0.984±0.012 | 0.987±0.004 | 0.824±0.014 | 0.979±0.004 | 0.955±0.024 | 0.910±0.042 |
|  | **TSS** | 0.923±0.012 | 0.980±0.013 | 0.953±0.038 | 0.957±0.038 | 0.922±0.018 | 0.649±0.029 | 0.876±0.021 | 0.883±0.050 | 0.821±0.083 |
| **East group** | **KAPPA** | 0.922±0.014 | 0.985±0.016 | 0.917±0.077 | 0.911±0.099 | 0.926±0.044 | 0.800±0.032 | 0.845±0.042 | 0.790±0.108 | 0.859±0.023 |
|  | **AUC** | 0.995±0.002 | 1.000±0.001 | 0.988±0.015 | 0.984±0.018 | 0.993±0.005 | 0.883±0.020 | 0.980±0.008 | 0.964±0.025 | 0.922±0.039 |
|  | **TSS** | 0.959±0.015 | 0.995±0.006 | 0.967±0.036 | 0.971±0.034 | 0.957±0.039 | 0.765±0.040 | 0.889±0.028 | 0.921±0.046 | 0.835±0.057 |
| **West group** | **KAPPA** | 0.919±0.036 | 0.995±0.010 | 0.972±0.056 | 0.856±0.125 | 0.978±0.030 | 0.808±0.047 | 0.895±0.059 | 0.841±0.117 | 0.936±0.069 |
|  | **AUC** | 0.996±0.003 | 1±0.000 | 0.994±0.014 | 0.975±0.022 | 0.998±0.003 | 0.870±0.024 | 0.991±0.007 | 0.972±0.024 | 0.960±0.053 |
|  | **TSS** | 0.966±0.017 | 0.998±0.003 | 0.987±0.028 | 0.950±0.045 | 0.992±0.012 | 0.741±0.049 | 0.952±0.023 | 0.939±0.049 | 0.921±0.106 |
